# Supplementary material for: Sasso Pisano Geothermal Field Environment Harbours Diverse Ktedonobacteria Representatives and Illustrates Habitat-Specific Adaptations
Source: Microorganisms. 2021 Jun 29;9(7):1402. doi: 10.3390/microorganisms9071402 (PMC8306680; doi:10.3390/microorganisms9071402)
Supplement: Supplementary file 1 [file microorganisms-09-01402-s001.zip › microorganisms-1274153-SI.pdf]

Supplementary Information

Sasso Pisano Geothermal Field Environment Harbors Diverse *Ktedonobacteria* Representatives and Illustrates Habitat-specific Adaptations

Sania Arif [1,*], Corinna Willenberg [1], Annika Dreyer [1], Heiko Nacke [2] and Michael Hoppert [1]

[1] Institute of Microbiology and Genetics, Department of General Microbiology, University of Göttingen, Grisebachstraße 8, 37077 Göttingen, Germany

[2] Institute of Microbiology and Genetics, Department of Department of Genomic and Applied Microbiology, University of Göttingen, Grisebachstraße 8, 37077 Göttingen, Germany

* Correspondence: Email: sarif@gwdg.de, Phone: +49-551-39-9657, Fax: +49-551-39-3808

Supplementary Information

Table S1. Complete details of the sampling sites

	Type	pH	Temperature	Source 1
Sp1	Yellowish brownish biofilm	5 – 6	29.7	Biofilm just above hot spring A vent
SP2	Yellowish-beige precipitates on rocks	-	25.6	Nearby the hot spring A
Sp11	Grey greenish discharge	7.4	98.9	Hot spring B vent
Sp12	Grey reddish mud	-	70.3	Around the hot spring B outlet
Sp25	Blackish muddy water	6.7	99.1	Hot spring C
Sp26	Blackish muddy discharge		75.4<br>56	20cm away hot spring C
Sp27	Brownish sediment and water			60cm away downstream spring C
Sp38	Leaves mixed with black discharge		40.5	Downstream hot spring D
Sp310	White discharge	6.2	67.8	Hot Spring D outlet
Sp513-15, Sp517, Sp719, Sp819-21, Sp3 Sp122	Soil samples		20-28	All over
Sp4	Water with greenish biomass	7.1	15	Water stream
Sp5	Greenish biofilm	2.0	45-55	Fumarole

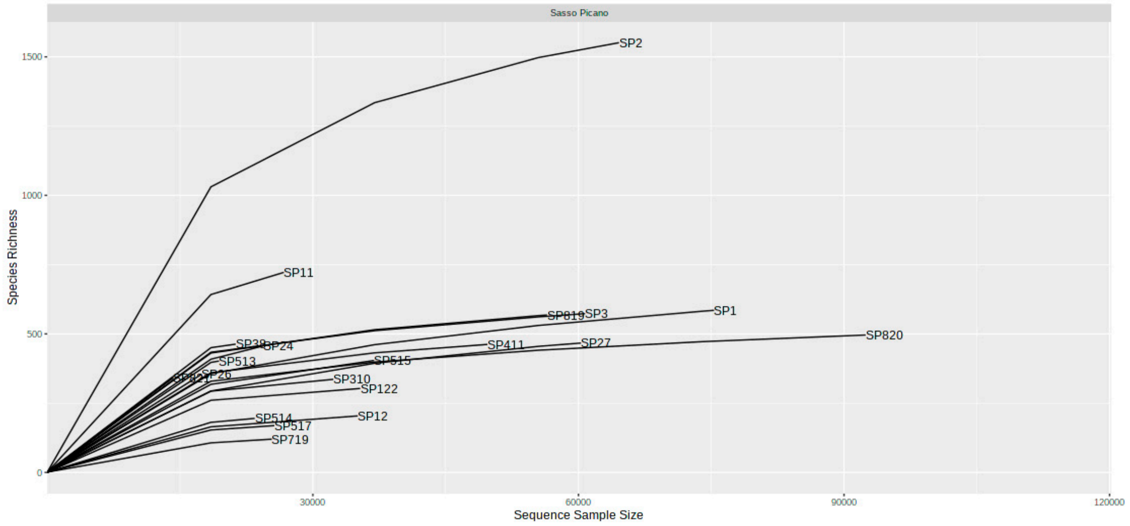

Figure S1. Rarefaction curves comparing species richness (Number of OTUs) and sequence sample size (number of reads) for all samples as indicated.

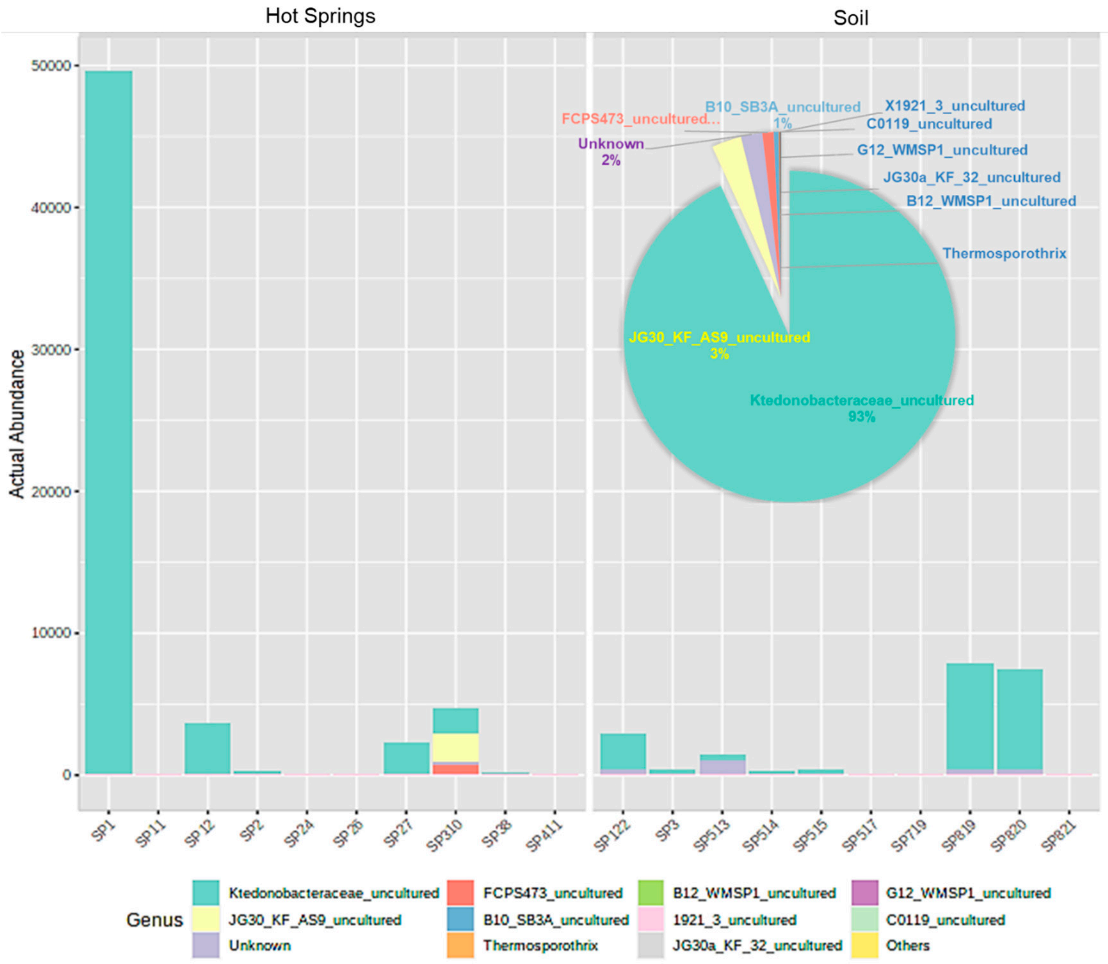

Figure S2. Number of sampled reads of the *Ktedonobacteria* members. Insert shows the relative abundance of the *Ktedonobacteria* at the genus level from all samples.

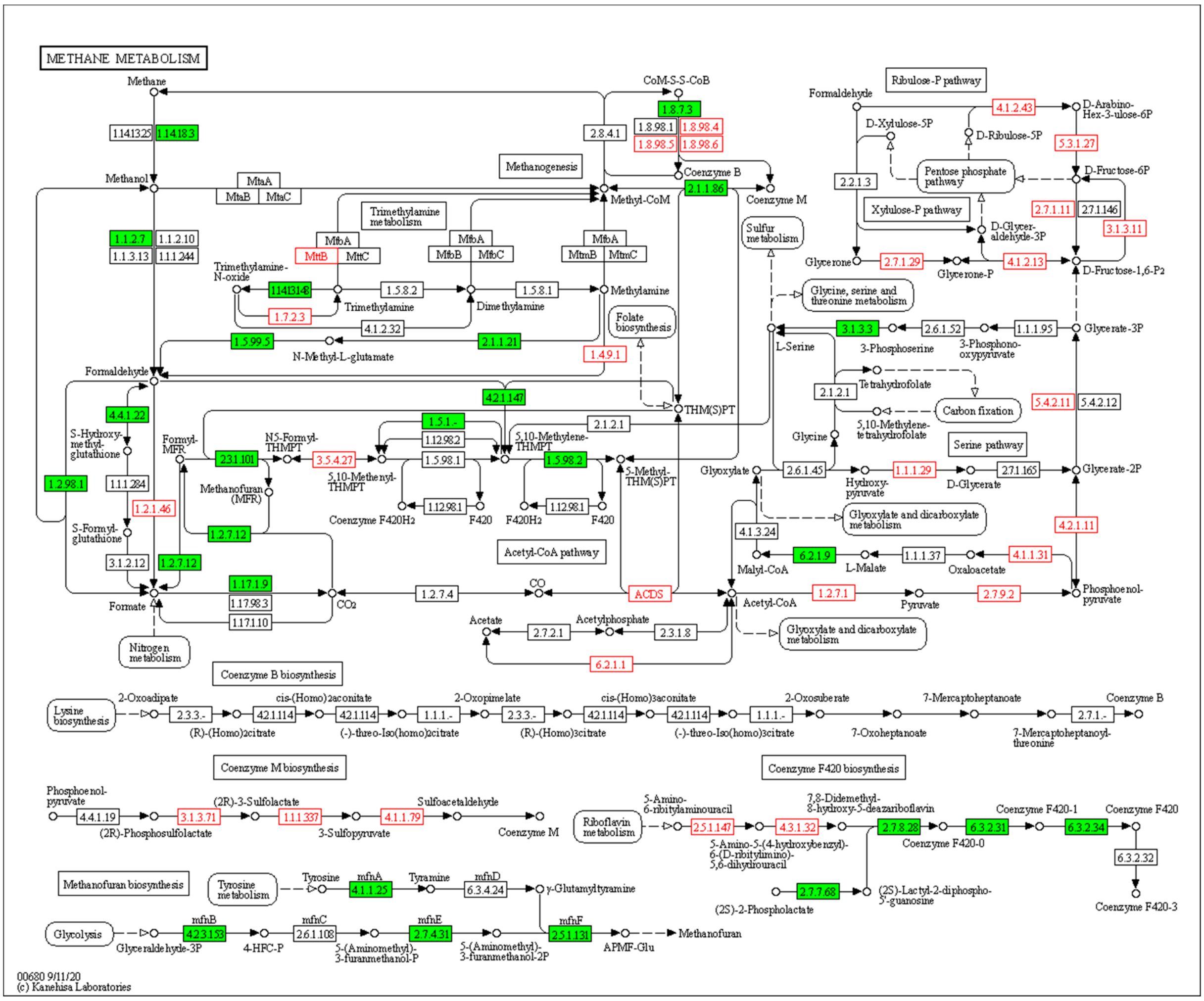

Figure S3. Differentially abundant genes involved in the methane metabolism. The differentially abundant genes KO numbers were mapped on the KEGG methane pathway map for hot springs (red) and soil (green) microbiome. The hot spring microbiome was found to be abundant in the genes involved in the anaerobic methane degradation as compared to the aerobic methane degradation in the soil microbiome.

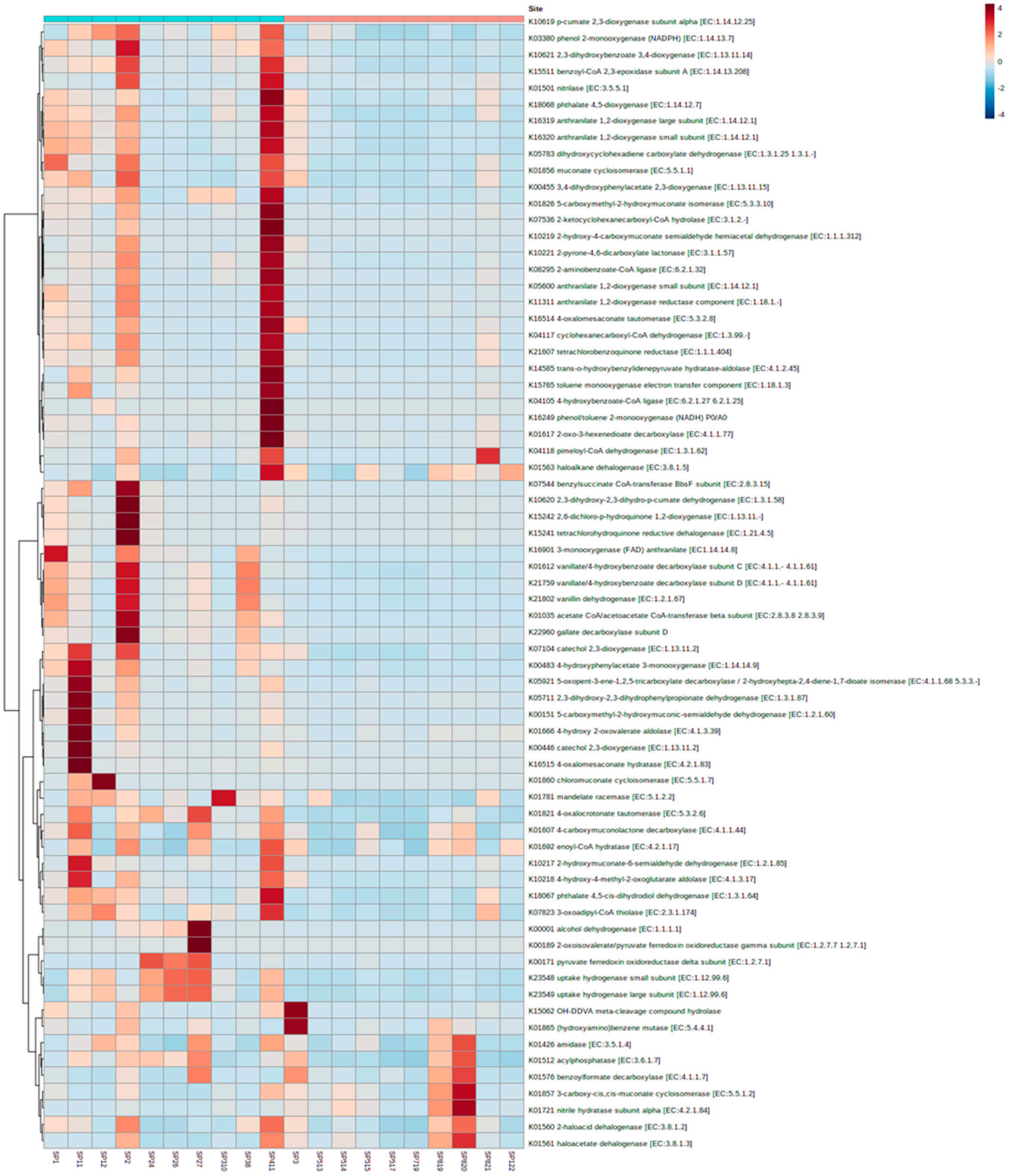

Figure S4. Relative abundance of differentially abundant genes involved in the aromatic compounds metabolism.

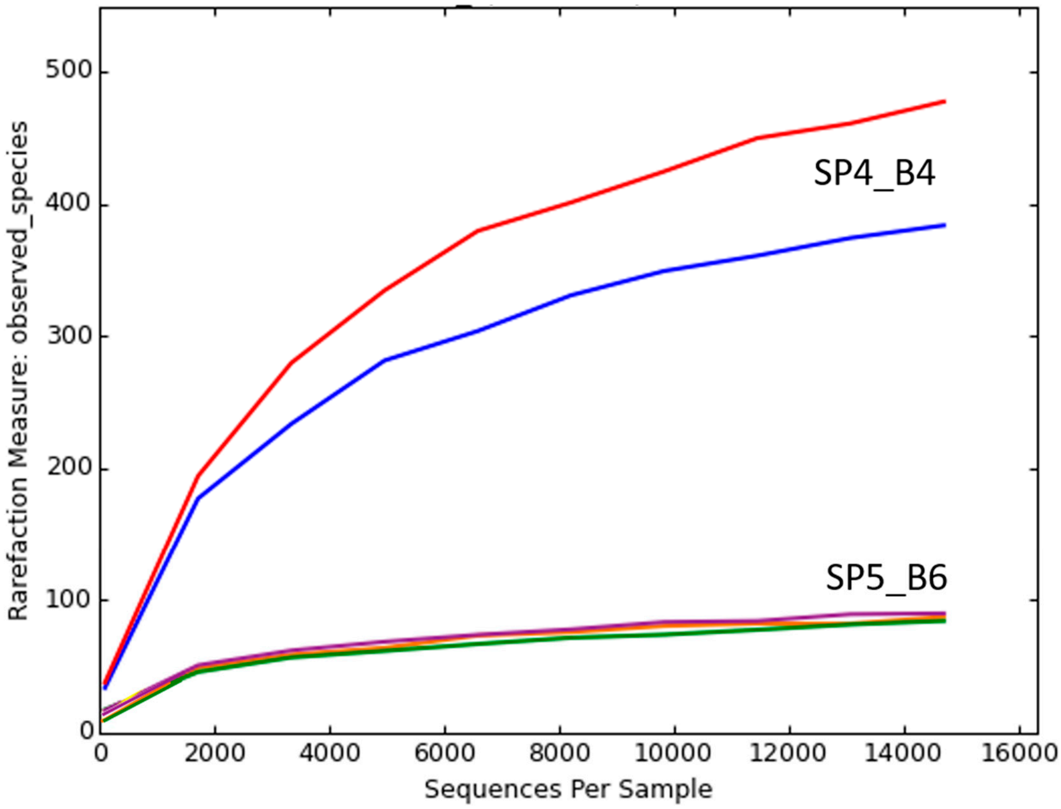

Figure S5. Eukaryotic rarefaction curve of a hot steam fumarole and neutral water stream. The number of OTUs are plotted against the reads on the x-axis. The extreme site SP5 samples OTUs have raised very quickly between 0 and 2500 reads and has almost reached saturation from 10,000 reads while the SP4 samples OTUs have a flatter rise.
